# Contrasting Properties of Gene-Specific Regulatory, Coding, and Copy Number Mutations in *Saccharomyces cerevisiae*: Frequency, Effects, and Dominance

**DOI:** 10.1371/journal.pgen.1002497

**Published:** 2012-02-09

**Authors:** Jonathan D. Gruber, Kara Vogel, Gizem Kalay, Patricia J. Wittkopp

**Affiliations:** 1 Department of Ecology and Evolutionary Biology, University of Michigan, Ann Arbor, Michigan, United States of America; 2 Department of Molecular, Cellular, and Developmental Biology, University of Michigan, Ann Arbor, Michigan, United States of America; University of Washington, United States of America

## Abstract

Genetic variation within and between species can be shaped by population-level processes and mutation; however, the relative impact of “survival of the fittest” and “arrival of the fittest” on phenotypic evolution remains unclear. Assessing the influence of mutation on evolution requires understanding the relative rates of different types of mutations and their genetic properties, yet little is known about the functional consequences of new mutations. Here, we examine the spectrum of mutations affecting a focal gene in *Saccharomyces cerevisiae* by characterizing 231 novel haploid genotypes with altered activity of a fluorescent reporter gene. 7% of these genotypes had a nonsynonymous mutation in the coding sequence for the fluorescent protein and were classified as “coding” mutants; 2% had a change in the *S. cerevisiae TDH3* promoter sequence controlling expression of the fluorescent protein and were classified as “*cis*-regulatory” mutants; 10% contained two copies of the reporter gene and were classified as “copy number” mutants; and the remaining 81% showed altered fluorescence without a change in the reporter gene itself and were classified as “*trans*-acting” mutants. As a group, coding mutants had the strongest effect on reporter gene activity and always decreased it. By contrast, 50%–95% of the mutants in each of the other three classes increased gene activity, with mutants affecting copy number and *cis*-regulatory sequences having larger median effects on gene activity than *trans*-acting mutants. When made heterozygous in diploid cells, coding, *cis*-regulatory, and copy number mutant genotypes all had significant effects on gene activity, whereas 88% of the *trans*-acting mutants appeared to be recessive. These differences in the frequency, effects, and dominance among functional classes of mutations might help explain why some types of mutations are found to be segregating within or fixed between species more often than others.

## Author Summary

Genetic dissection of phenotypic differences within and between species has shown that mutations affecting either the expression or function of a gene product can contribute to phenotypic evolution; mutations that alter gene copy number have also been shown to be an important source of phenotypic variation. Predicting when and why one type of mutation is more likely to underlie a phenotypic change than another remains a pressing challenge for evolution biology. Understanding the relative frequency and properties of different types of mutations will help resolve this issue. To this end, we isolated 231 mutants with altered activity of a focal gene. Mutants were classified into one of four functional classes (i.e., coding, *cis*-regulatory, *trans*-acting, or copy number) based on the location and nature of mutation(s), or lack thereof, within the focal gene. Mutant effects on focal gene activity were assessed in both haploid and diploid cells. These data identified differences in the frequency, effects, and dominance (relative to the wild-type allele) among functional classes of mutants that help explain patterns of genetic variation within and between species.

## Introduction

Mutations are the ultimate source of genetic variation, thus understanding the properties of new mutations is important for both medical and evolutionary genetics. Large-scale sequencing surveys have recently measured mutation rates for different types of DNA lesions (e.g., transitions, transversions, indels, rearrangements, duplications) in a variety of organisms [1]–[3], but little remains known about the genetic properties of these mutations or their effects on the activity of individual genes. Although not often incorporated into population genetic models of the evolutionary process, differences in the frequency and properties of different types of mutations can influence evolutionary paths [4]–[6].

From the perspective of a single gene, mutations affecting its activity can be divided into four functional classes: [nonsynonymous] coding mutations that alter the sequence of the encoded RNA or protein gene product, *cis*-regulatory mutations that alter (typically, non-coding) sequences that regulate the gene's expression in an allele-specific manner, *trans*-acting mutations that alter coding or *cis*-regulatory sequences of other genes in the genome and affect activity of the focal gene via a diffusible gene product, and copy number mutations resulting from duplications or deletions that change the number of copies of the focal gene in the genome. As the raw material of evolutionary change, all of these types of mutations have the potential to become polymorphisms segregating at an appreciable frequency within a species and/or substitutions fixed between species, yet studies identifying the genetic basis of trait differences suggest that some types of changes underlie phenotypic differences more often than others (reviewed by [7]–[9]).

The apparent inequality in the contribution of different types of mutations to phenotypic evolution is often explained by invoking differences in pleiotropy (i.e., the number of traits affected by a mutation) among functional classes. Increased pleiotropy is assumed to increase the chance that a mutation has deleterious effects on fitness and will be disfavored by natural selection. One example of this is that coding mutations are commonly expected to be more pleiotropic (and hence have lower average fitness) than *cis*-regulatory mutations ([7], [9], [10], but see [11]). Although undoubtedly important, pleiotropy is only one factor influencing the probability that a certain type of mutation is fixed. For example, the direction and magnitude of a mutation's effect on gene activity and whether or not the mutation is dominant to the wildtype allele are also expected influence the evolutionary trajectories of new mutations in diploid populations. Of course, these factors matter only after a mutation has occurred, thus mutation rates can influence the evolutionary process as well [7], [12]–[14]. The frequency, effects, and dominance of new mutations have all been predicted to vary among functional classes of mutations [15]–[17], but little data has been available to test these predictions [13], [15].

To directly compare these parameters among functional classes of mutations, we systematically isolated and quantitatively characterized over 200 mutations in *Saccharomyces cerevisiae* affecting activity of a focal gene. To make this experiment feasible, we used a mutagen to elevate the mutation rate and studied mutations affecting activity of a reporter gene expressing Yellow Fluorescent Protein (YFP) that could be scored quantitatively in thousands of living cells per second using flow cytometry. Expression of this heterologous fluorescent protein was controlled by native *S. cerevisiae* promoter and terminator sequences (which allowed us to interrogate endogenous *S. cerevisiae* transcriptional regulatory networks) and the mutagen was expected to cause mutations relatively uniformly across the genome.

Using this experimental system, we measured the proportion of cells with new mutations that altered activity of the reporter gene and used this proportion to estimate the spontaneous mutation rate for this phenotypic change. We then isolated 231 mutants with altered activity of the reporter gene and subjected them to further characterization, including determining the relative frequency of different types of mutations, comparing their effects on reporter gene activity, and assessing their dominance relative to the wildtype allele. These data revealed differences in the frequency, effects, and dominance among coding, *cis*-regulatory, *trans*-acting, and copy number mutations that are expected to influence the relative contribution of different types of mutations to phenotypic variation within and between species.

## Results

**Figure 1:**
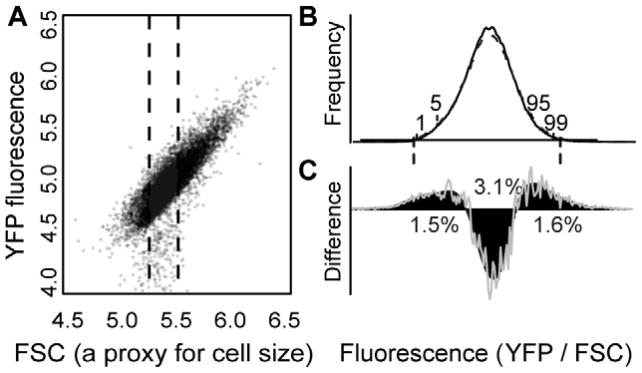
EMS treatment increased the frequency of cells with extreme YFP fluorescence. (A) The relationship between “forward scatter” (“FSC”, a proxy for cell size) and YFP fluorescence is shown for a population of control cells. FSC and YFP fluorescence are both reported in arbitrary units on a log scale. A similar linear relationship was observed for other genotypes. Approximately 2% of flow cytometry “events” had YFP fluorescence less than the range plotted and are not shown. The fluorescence phenotype of each sample in the secondary screen was calculated as the median YFP/FSC ratio for FACS events with FSC values between 5.30 and 5.55 (indicated with dotted lines). (B) The distribution of YFP fluorescence phenotypes is shown for EMS-treated (dashed curve) and control cells (solid curve) from one of the nine replicate populations of EMS-treated and control cells analyzed in the primary screen. Locations of the 1st, 5th, 95th, and 99th percentiles of the control sub-population are indicated, and vertical dashed lines show the average thresholds used for cell sorting (see also Table S1). (C) The difference between the number of EMS-treated and control cells in the population is plotted for a range of fluorescence levels (grey line). The black curve shows a spline fit to these data. Positive values indicate fluorescence phenotypes that were more abundant in the EMS-treated sample, whereas negative values indicate fluorescence phenotypes that were more abundant in the control sample. The spline crosses zero at approximately the 17th and 84th percentiles of the control population. The percentage of the EMS-treated population that is either over or under represented is shown for the following percentile ranges: 1–17, 17–84, 84–99. The X-axis representing YFP fluorescence levels has the same scale as in panel B.

To characterize the spectrum of mutations affecting activity of a focal gene, we screened mutagenized cells containing a fluorescent reporter gene and quantified cellular fluorescence using flow cytometry. Mutagenesis was performed using ethyl methanesulfonate (EMS), and the increase in mutation rate was controlled by titrating exposure of cells to this chemical. The reporter gene was constructed by fusing the coding sequence of the Venus variant [18] of YFP to the *S. cerevisiae CYC1* terminator [19], and placing them both under the control of 5′ intergenic sequence of the *S. cerevisiae TDH3* gene. This chimeric transgene (*P_TDH3_-YFP*) was integrated into a pseudogene on the first chromosome of *S. cerevisiae*, where integration of fluorescent reporter genes has previously been found to have no measurable effect on fitness (B. Williams, personal communication). For each cell, the activity of *P_TDH3_-YFP* was measured as YFP fluorescence per unit of “forward scatter” (FSC); FSC is proportional to cell size [20] and is linearly related to YFP fluorescence (Figure 1A). In the absence of amino acid changes in YFP, cellular fluorescence is expected to be linearly related to YFP protein abundance, as has been shown for the related Green Fluorescent Protein [21].

### A spontaneous mutation rate affecting *P_TDH3_-YFP* activity was estimated from an EMS-treated population

To determine the frequency of mutations that affected *P_TDH3_-YFP* activity, which is expected to reflect the genome-wide mutational target size for this phenotype, we measured YFP fluorescence in each cell of a mixed sample containing both EMS-treated and untreated cells. Cells that were not exposed to EMS were considered control cells and labelled with Cy5 (a fluorescent dye), but otherwise processed identically to the EMS-treated cells. Comparing YFP fluorescence between >25,000 control cells labelled with Cy5 and >20,000 unlabelled control cells showed that Cy5 labeling had no significant effect on the measurement of YFP fluorescence phenotypes (P = 0.9, t-test).

The EMS-treated population displayed an approximately equal increase of cells with YFP fluorescence levels in both tails of the distribution (Figure 1B, 1C), suggesting that mutations increasing and decreasing fluorescence occurred at similar rates. The increase in cells with both high and low fluorescence was taken as the frequency (*f = 0.0298*) of EMS-induced mutants with altered activity of *P_TDH3_-YFP*, whereas the remaining cells were assumed not to carry any mutations affecting *P_TDH3_-YFP* activity. Assuming mutations affecting *P_TDH3_-YFP* activity were Poisson distributed, the inferred frequency of genotypes without a relevant mutation (*P_0_* = *1−f = 0.9702*) suggested an average of 0.0303 mutations affecting *P_TDH3_-YFP* activity per genome in the EMS-treated cells. Given this mutation rate, a Poisson process predicts that 2.94% of the EMS-treated cells should have exactly one relevant mutation (*P_1_*), whereas just 0.04% of the EMS-treated cells (1% of all mutants) should have more than one mutation affecting *P_TDH3_-YFP* activity (*P_>1_*).

To estimate a spontaneous mutation rate for *P_TDH3_-YFP* activity from this mutagen-treated population, we measured the frequency of canavanine resistance mutants in the same EMS-treated population, and found that it was 5737-fold higher than the spontaneous canavanine resistance mutation rate reported by [22]. Assuming that a similar proportion of sites affecting canavanine susceptibility and *P_TDH3_-YFP* activity were targeted by EMS, our data suggest a spontaneous mutation rate for quantitative changes in *P_TDH3_-YFP* activity of 0.0303/5737 or 5.3×10^−6^ per haploid genome per generation (Text S1). An alternative estimate of the spontaneous mutation rate, based on the number of empirically confirmed mutants in each tail of the EMS-treated distribution (described in the next section), was also calculated and is presented in Table S4.

### A collection of 231 mutants affecting *P_TDH3_-YFP* activity was established using fluorescence activated cell sorting (FACS)

To isolate individual cells with abnormal *P_TDH3_-YFP* activity for further characterization, cells exhibiting YFP fluorescence less than a minimum threshold near the 1st percentile of control cells and greater than a maximum threshold near the 99th percentile of control cells (Figure 1B) were collected using FACS. Exact sorting thresholds for the nine replicate sorting experiments are shown in Table S1. On average, cells with YFP fluorescence similar to that of the lowest fluorescing 0.82% and highest fluorescing 0.64% of the control population were sorted. These threshold levels of YFP fluorescence resulted in sorting cells from the lowest fluorescing 1.21% and highest fluorescing 1.04% of the mutagenized population, suggesting that (1.21–0.82)/1.21, or 32.2%, of EMS-treated cells sorted from the low-fluorescence tail and (1.04–0.64)/1.04, or 38.5%, of EMS-treated cells sorted from the high-fluorescence tail were mutants. In all, 864 FACS “events” (*i.e.*, cells or other particles) were sorted from each tail of the EMS-treated subpopulation, and 864 FACS events were sorted from each tail of the control population, for a total of 3456 FACS events arrayed individually on solid media. The percentage of sorted events that formed colonies was similar between the EMS-treated and control populations (68% vs 70%, P = 0.26, Fisher's Exact test), suggesting that EMS-induced mutations severely limiting growth were rare among cells sorted from this population. A slightly larger, and statistically significant, difference was observed, however, between the percentage of sorted events from the high- and low-fluorescing tails that formed colonies for both EMS-treated and control cells (65% vs 70% for mutagenized cells, and 67% vs 72% for control cells; P = 0.03 in each comparison, Fisher's Exact Test). The similar asymmetry observed in the mutagenized and control populations suggests that it was not caused by the EMS treatment.

Each colony was used to inoculate a liquid culture, and YFP fluorescence was measured in at least 5,000 cells from each of these clonal cultures by flow cytometry. The YFP fluorescence phenotype of each culture was calculated as the median YFP/FSC ratio of all cells within a fixed range of FSC values (Figure 1A). To determine the effect on *P_TDH3_-YFP* activity of any mutation(s) present in a recovered genotype, we calculated the difference in YFP fluorescence between each genotype and the mean YFP fluorescence of replicate control cultures, and then divided it by the standard deviation of YFP fluorescence phenotypes among the replicate control cultures. This value is a test statistic known as a Z-score (Z), and reflects the magnitude and direction of each genotype's effect on YFP fluorescence relative to the starting (unmutagenized) genotype as well as the likelihood that this effect is significantly different from 0. Given our experimental design, only mutations that prevented colony formation on solid media, slowed growth in liquid culture enough to preclude sampling 5,000 cells, or had effects on YFP fluorescence below our detection limits should have been systematically eliminated from our collection.

Genotypes isolated from the EMS-treated population with |Z|>2.58 were considered mutants and subjected to further analysis. This statistical threshold corresponds to a 99% confidence interval for the mean of the control population, and implies that all genotypes considered mutants showed a change in YFP fluorescence supported by a [two-tailed] p-value<0.01. On the basis of this statistical cut-off, 231 (22%) of the 1064 liquid cultures derived from the EMS-treated colonies were considered mutants (Table S2). By contrast, only 16 (1%) of the 1137 cultures derived from the control colonies exceeded the |Z| = 2.58 significance threshold (Table S2); these 16 isolates are not included in the collection of mutants discussed below. In addition to these changes in median YFP fluorescence, 18.6% of EMS-treated genotypes classified as mutants, 4.4% of EMS-treated genotypes not classified as mutants, and 1.8% of genotypes isolated from the control population showed significant changes in the variance of YFP fluorescence (Figure S1). Although changes in both the median and variance of YFP fluorescence might or might not be caused by the same mutation, the elevated proportion of genotypes with altered variance among EMS-treated genotypes classified as YFP fluorescence mutants suggests that they might often be one and the same.

### Frequency of mutants affecting *P_TDH3_-YFP* activity differed among mutational classes

Mutants affecting *P_TDH3_-YFP* activity were identified solely on the basis of their YFP fluorescence phenotype, thus we expected them to include genotypes with (1) mutations in the coding sequence of *P_TDH3_-YFP*, (2) mutations in *cis*-acting sequences of *P_TDH3_-YFP*, (3) mutations outside of the known *cis*-regulatory and coding sequences of *P_TDH3_-YFP* that putatively have *trans*-acting effects on the cell's YFP fluorescence phenotype, and (4) duplications or deletions changing the copy number of *P_TDH3_-YFP* (copy number variants, CNVs).

**Figure 2:**
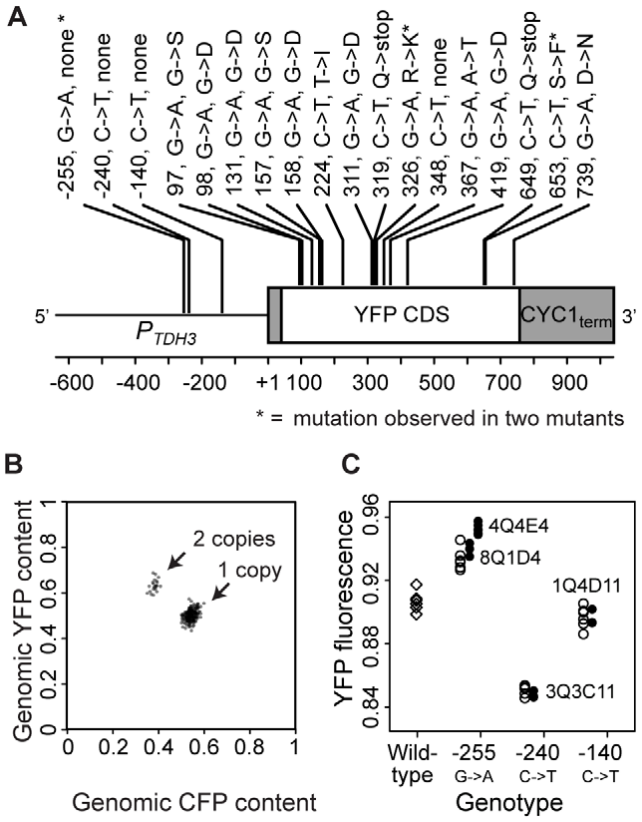
Mutations in the coding and promoter sequences of *P_TDH3_-YFP* as well as changes in its copy number were found among the 231 mutant genotypes. (A) The schematic depicts the *P_TDH3_-YFP* reporter gene and is drawn to scale. Annotations indicate the location of each mutation relative to the transcription start site (+1) of *TDH3* [68], the nucleotide substitution observed, and the change in amino acid sequence if applicable. Asterisks indicate mutations observed in two mutant genotypes. Mutations at positions −255, −240 and −140 are located within the promoter, and the mutation at position 348 is a synonymous change. (B) In 221 of the 231 mutants, the relative copy number of *P_TDH3_-YFP* and *P_TDH3_-CFP* was determined by pyrosequencing. Clusters of points representing genotypes with one copy of *P_TDH3_-YFP* (CFP and YFP≈1/2) and two copies of *P_TDH3_-YFP* (CFP≈1/3 and YFP≈2/3) are indicated with arrows. (C) Median YFP fluorescence is plotted for each of the replicate populations analyzed for eight distinct genotypes: unmutagenized cells containing the “wildtype” *P_TDH3_-YFP* sequence (diamonds); each of the four regulatory mutant genotypes (8Q1D4, 4Q4E4, 3Q3C11, and 1Q4D11 in Table S3) that contained a promoter mutation (filled circles); and genotypes in which one of the promoter mutations (−255, −240, or −140) was introduced into same genetic background as the wildtype *P_TDH3_-YFP* gene (open circles). Populations of cells containing any one of the promoter mutations showed a significant change in YFP fluorescence relative to the wildtype genotype (*P_−255_* = 0.002, *P_−240_* = 0.002, *P_−140_* = 0.015, MWW test). The mutation at −255 had an effect on YFP fluorescence equivalent to that of mutant genotype 8Q1D4 (*P* = 0.261, MWW), but not to that of mutant genotype 4Q4E4 (*P* = 0.0002, MWW). The effects on YFP fluorescence of mutations at −240 and −140 were equivalent to those of the mutant genotypes (3Q3C11 and 1Q4D11, respectively) that harbored them (*P_−240_* = 0.262 and *P_−140_* = 0.262, MWW).

To identify genotypes with mutations in the coding and *cis*-regulatory sequences of *P_TDH3_-YFP*, we sequenced the entire *P_TDH3_-YFP* transgene in each of the 231 mutants. 16 independently isolated genotypes were each found to contain a mutation predicted to change an amino acid or introduce a stop codon in YFP; two of these observed mutations were found in two genotypes each (Figure 2A). Additionally, one mutant was found to contain a synonymous mutation within the YFP coding region (Figure 2A). Four mutants had mutations within the *cis*-regulatory promoter region of *P_TDH3_-YFP*, two of which carried the same mutation (Figure 2A). None of the 231 mutants had a mutation in the *CYC1* terminator (Figure 2A), nor did any contain more than one mutation in the entire *P_TDH3_-YFP* gene (i.e., *TDH3* promoter, *YFP* coding sequence and *CYC1* terminator). Cases where the same mutation was found in two mutants could have resulted from recurrent mutation or common ancestry, although the experiment was designed to minimize the potential for recovering clonally related mutants (see Text S1) and in at least one case (described below) the shared mutation exists on different genetic backgrounds, suggesting independent origins.

EMS is not generally thought to induce changes in copy number [23], [24], but spontaneous duplications are common in *S. cerevisiae* [1]. Therefore, we tested for changes in the copy number of *P_TDH3_-YFP* by mating each haploid mutant to a closely related genotype (of the opposite mating type) in which the YFP coding sequence in *P_TDH3_-YFP* was replaced with the coding sequence for a Cyan Fluorescent Protein (CFP, 95% amino acid sequence identity with YFP [25]). Pyrosequencing was then used to compare the relative frequency of YFP and CFP alleles in genomic DNA extracted from each of the resulting diploid genotypes. 22 (10%) of the 221 mutants tested showed evidence of *P_TDH3_-YFP* duplications (Figure 2B); 10 mutants were not analyzed because they either failed to produce diploids or showed evidence of contamination. 16 genotypes isolated from the control population with |Z|>2.58 were also tested, and 5 (31%) showed evidence of more than one copy of *P_TDH3_-YFP*. This high frequency of copy number variants in the control population is consistent with the idea that copy number variants in the EMS-treated population also resulted primarily from spontaneous duplications. This in turn suggests that duplications are the most common type of spontaneous mutation affecting *P_TDH3_-YFP* activity, given that we estimated the frequency of point mutations was elevated ∼5700-fold by EMS in our screen.

On the basis of these data, we divided the 221 mutants tested for copy number variation into four classes (Table S3): the 16 mutants containing a mutation affecting the amino acid sequence of YFP were classified as “coding”; the 22 mutants containing a duplication of *P_TDH3_-YFP* were classified as copy number variants, or “CNV”s; the 4 mutants containing a mutation in the *TDH3* promoter were classified as “*cis*-regulatory”; and the 179 mutants that had neither a *cis*-regulatory or non-synonymous mutation in *P_TDH3_-YFP* nor a change in its copy number were classified as “*trans-*acting”. This large *trans*-acting class of mutants is expected to include coding and noncoding changes in genes other than *P_TDH3_-YFP* that regulate its transcription and post-transcriptional processing as well as mutations that impact elements of the cell that affect fluorescence per unit cell size (i.e., FSC) (e.g., pH [26]). Epigenetic changes are also possible. Of the 10 mutant genotypes that we were unable to test for *P_TDH3_-YFP* copy number, none showed any sequence differences in *P_TDH3_-YFP*, indicating that they could be either CNVs or *trans-*acting mutants (Table S3). Because of this ambiguity, these 10 genotypes were excluded from the comparisons among mutant classes described below.

Mutations affecting the amino acid sequence of YFP or the number of copies of *P_TDH3_-YFP* were assumed to explain the mutant phenotypes of genotypes in which they occur; however, we were less confident of this assumption for the promoter mutations. Therefore, we empirically tested whether each of the three mutations identified in the promoter region was (1) sufficient to alter YFP fluorescence and (2) sufficient to recreate the YFP fluorescence phenotype of the mutant genotype(s) that harbored it. Site-directed mutagenesis was used to introduce each mutation into the ancestral (unmutagenized) genotype, and YFP fluorescence was analyzed in a haploid population of these genetically modified cells using flow cytometry. In all three cases, populations of cells containing one of these promoter mutations showed in a significant change in YFP fluorescence relative to cells with the ancestral promoter (P<0.05, Mann-Whitney-Wilcoxon (MWW); Figure 2C). For three of the four genotypes containing a promoter mutation, this mutation was sufficient to recapitulate the change in YFP fluorescence (Figure 2C), showing that the promoter mutation was solely responsible for the observed mutant phenotype. The one exception was a genotype that carried the same promoter mutation as another strain; in this case, the promoter mutation only partially recreated the mutant's YFP fluorescence (Figure 2C), indicating that this genotype (mutant 4Q4E4) contained more than one mutation affecting YFP fluorescence.

### Effects of mutations on *P_TDH3_-YFP* activity differed among mutational classes

As described above, the Z-score calculated for each sorted genotype describes the magnitude and direction of its effects on *P_TDH3_-YFP* activity relative to the control (unmutagenized) genotype. To determine the relative frequency of mutations that increased and decreased YFP fluorescence, we examined the sign of the Z-score for each of the 231 mutants with |Z|>2.58 and found that 162 (70.1%) showed increased fluorescence (Z>0). When alternative thresholds of either |Z|>1.96 or |Z|>1.645, corresponding to p<0.05 and p<0.1, respectively, were used to identify mutants, 68% showed increased fluorescence. This excess of mutants with increased YFP fluorescence was surprising given the similar increases in cell number observed in both tails of the full EMS-treated population (Figure 1B, 1C). As described above, differences in colony formation rates were observed between the high- and low-fluorescing tails; however, they are unlikely to explain the apparent excess of high-fluorescing mutants: 65.3% of FACS events sorted from the low-fluorescing tail formed colonies and 38.5% of this group were expected to be mutants, whereas 70.1% of FACS events sorted from the high-fluorescing tail formed colonies and 32.2% of these were expected to be mutants, suggesting that about half (52.7%) of recovered mutants should increase fluorescence. This discrepancy might instead result from a nonuniform distribution of mutants within each tail, such that sorting from a slightly larger tail at the low-fluorescing end of the distribution (1.22% vs 1.04% of cells in the EMS-treated population) resulted in a lower proportion of sorted cells being classified as mutants.

**Figure 3:**
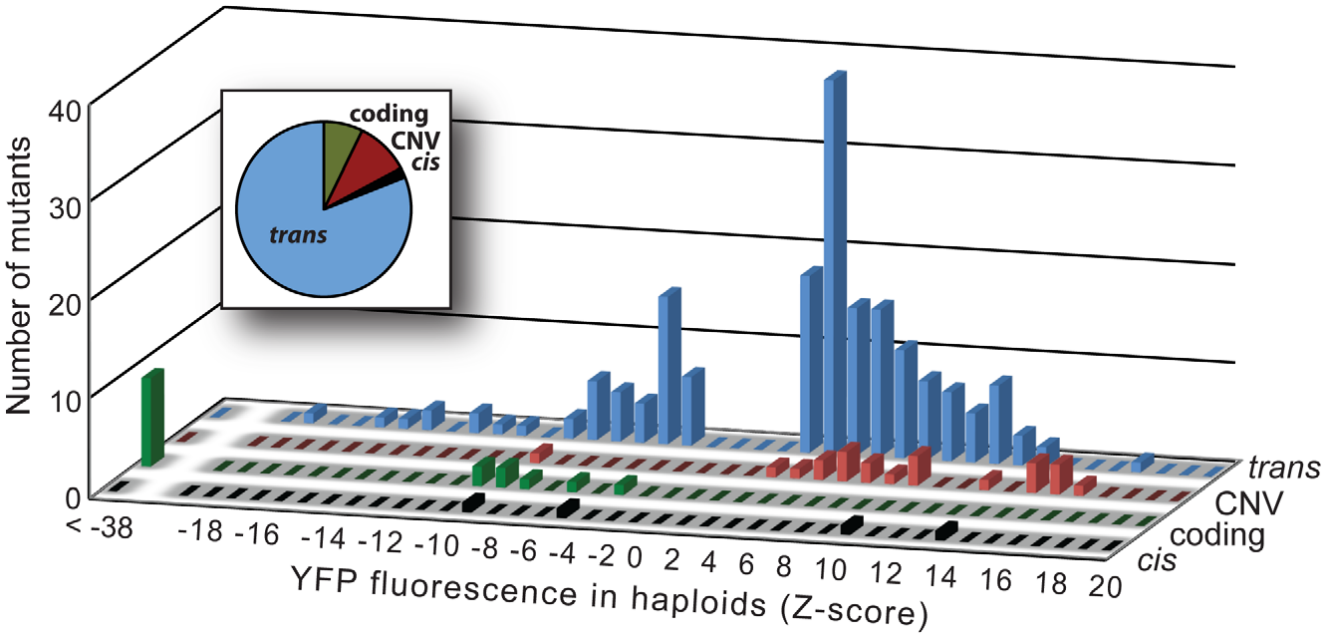
Effects on YFP fluorescence in haploid cells differ among mutational classes. The effects on YFP fluorescence of the 4 *cis*-regulatory (black), 16 coding (green), 22 CNV (red), and 179 *trans*-acting (blue) mutants are summarized in histograms. For each mutational class, the height of each bar indicates the number of mutants with the corresponding effect (as measured by Z-score) on YFP fluorescence in haploid cells. Positive Z-scores indicate increases in YFP fluorescence relative to control cells and negative Z-scores indicate decreases in YFP fluorescence relative to control cells. The relative frequency of mutants in each of the four mutational classes is also shown in the inset pie chart.

Comparing the distributions of Z-scores among the four mutational classes showed differences in both the magnitude and direction of effects among groups (Figure 3). For example, none of the 16 coding mutants increased fluorescence, compared to 21 (95%) of the 22 CNVs, 2 (50%) of the 4 *cis*-regulatory mutants, and 131 (73%) of the 179 *trans*-acting mutants. Considering only the magnitude of the change in YFP fluorescence caused by each mutant (|Z|), we found statistically significant pairwise differences among coding, CNV, and *trans-*acting mutants (P≤0.02 in all 3 comparisons, MWW test). With only four *cis*-regulatory mutants recovered from our screen, we had little power to detect differences in comparison with other classes, and all three pairwise tests involving this class failed to reach statistical significance (P≥0.15 in all cases, MWW test). Overall, coding mutants had the largest effect on gene activity (median |Z| = 48), followed by *cis*-regulatory mutants (median |Z| = 8.1) and CNVs (median |Z| = 8.0), and finally *trans-*acting mutants (median |Z| = 4.6).

### Dominance of mutations affecting *P_TDH3_-YFP* activity differed among mutational classes

We isolated mutants in haploid cells so that we could recover recessive mutations; however, wild populations of many eukaryotes, including *S. cerevisiae*, tend to be diploid. To determine how the haploid mutant genotypes in our collection act in diploid cells, we again crossed each mutant genotype with the reference strain containing *P_TDH3_-CFP* that was used to identify CNVs. YFP and CFP fluorescence was measured in at least 9,000 diploid cells for each mutant genotype using flow cytometry. Z-scores describing YFP and CFP fluorescence were calculated for each mutant by comparing their fluorescence to that of replicate populations of control diploid cells resulting from mating the ancestral, unmutagenized *P_TDH3_-YFP* haploid genotype to the reference haploid genotype containing *P_TDH3_-CFP*. We successfully tested all 16 coding mutants, all 4 *cis*-regulatory mutants, 21 of the 22 CNVs, and 171 of the 179 *trans*-acting mutants for their effects on YFP and CFP fluorescence in diploid cells.

**Figure 4:**
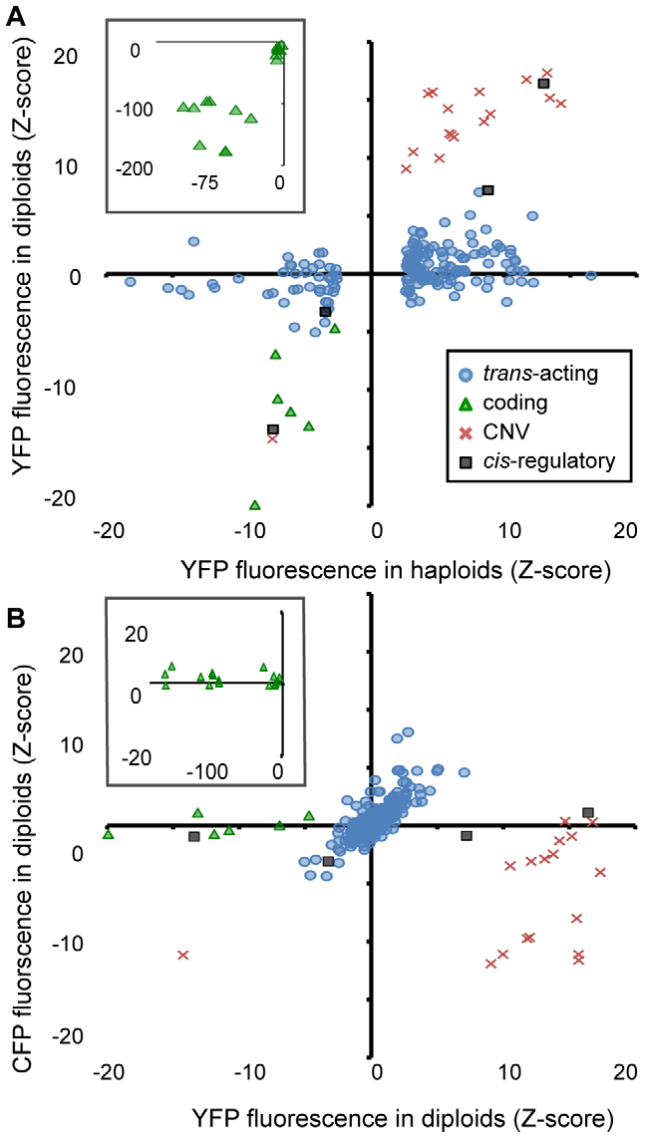
Effects of heterozygous mutant alleles on YFP and CFP fluorescence differ among mutational classes in diploid cells. (A) The effect of each mutant genotype on YFP fluorescence in haploid cells (X-axis) is compared to the effect of the heterozygous mutant genotype on YFP fluorescence in diploid cells (Y-axis). These diploid cells were heterozygous for the mutant *P_TDH3_-YFP* haploid genome and a reference haploid genome containing *P_TDH3_-CFP*. Black squares indicate *cis*-regulatory mutants, blue circles indicate *trans*-acting mutants, green triangles indicate coding mutants, and red crosses indicate CNV mutants. (B) Using the same symbols to represent the four mutational classes as in (A), the effect of each mutant on YFP (X-axis) and CFP (Y-axis) fluorescence in diploid cells is shown. Insets in (A) and (B) show only coding mutants and cover the larger ranges of Z-scores needed to plot all of the mutants in this class.

To assess the dominance of each mutant relative to the reference strain (i.e., its ability to affect *P_TDH3_-YFP* activity in heterozygous, diploid cells), we compared the Z-scores for YFP fluorescence of each mutant from haploid and diploid cells (Figure 4A). We found that the effects of coding, CNV, and *cis*-regulatory mutants in diploid cells (median |Z| = 96, 15, and 10, respectively) were more similar to their effects in haploid cells (regression coefficients (b) of 1.67, 1.50, and 1.34, respectively, from a model II regression) than were those of *trans*-acting mutants (median |Z| = 1, b = 0.12). These data show that as a group, the *trans*-regulatory mutants were much more recessive than mutants from any of the other classes. This was also seen using a threshold of |Z| = 2.58 to classify mutants as recessive (i.e., no significant effect on YFP fluorescence in diploids): none of the coding, CNV, or *cis*-regulatory mutants were called recessive, whereas 151 (88%) of the 171 *trans*-acting mutants tested were.

To determine whether a mutant genotype had similar effects on both alleles of the reporter gene present in diploid cells, we compared the effects of each genotype on [diploid] YFP and CFP fluorescence (Figure 4B). This analysis showed that CNVs had the largest effect on CFP fluorescence (median |Z| = 4.2 compared to median |Z| = 1 for all other mutant classes). Surprisingly, 15 of the CNVs showed a significant decrease in CFP fluorescence despite a significant increase in YFP fluorescence. Of the coding mutants tested, the majority (14 of 16) showed no significant effect on CFP fluorescence (|Z|<2.58), as expected. The remaining two showed small increases in CFP fluorescence (Z = 2.6 and 2.8, respectively) despite showing decreases in YFP fluorescence (Z = −167 and −28). These genotypes might harbor amino acid changes that alter the emission spectrum of the mutant YFP protein and cause it to overlap that of CFP. Mutants classified as *cis*-acting were also not expected to alter CFP fluorescence, and three of the four *cis*-acting mutants did not (|Z| = 0.75, 1.1, and 0.68). The one *cis*-regulatory mutant that showed an effect on CFP fluorescence (1Q4D11, whose mutant phenotype in haploids appeared to be caused solely by the identified promoter mutation, Figure 2C) decreased both CFP and YFP fluorescence (Z = −2.7, P = 0.006), suggesting transvection [27]. Finally, non-recessive mutants in the *trans*-acting class were expected to have similar effects on both YFP and CFP fluorescence in diploid cells, and 13 (65%) of the 20 *trans*-acting mutants with |Z|>2.58 for YFP fluorescence in diploid cells also showed a significant (|Z|>2.58) effect on CFP fluorescence in the same direction. Considering all mutants in all classes, the relationship between CFP and YFP fluorescence in diploid cells was strongest for the *trans*-acting mutant class (Figure 4B; b = 1 compared with b = 0.00, 0.37, and 0.08 for coding, CNV, and *cis*-regulatory mutants, respectively).

## Discussion

This study provides a systematic survey and functional analysis of mutations affecting activity of a reporter gene (*P_TDH3_-YFP*) in *S. cerevisiae*. By comparing unmutagenized and mutagenized subpopulations of a clonal culture, we estimated a spontaneous mutation rate for activity of *P_TDH3_-YFP* of 5.3×10^−6^ per haploid genome per generation, which, in *S. cerevisiae*, is intermediate between spontaneous mutation rates reported for single gene loss-of-function phenotypes (10^−6^–10^−8^, [22], [28]–[32] and more complex organismal phenotypes such as growth rate (10^−3^, [33]), and suggests that *P_TDH3_-YFP* activity is controlled by a moderate number of genes. Further characterization of 231 mutants with changes in *P_TDH3_-YFP* activity revealed differences in the relative frequency, effects, and dominance of different types of mutations (Table 1) that are expected to influence their likelihood of contributing to phenotypic evolution.

**Table 1:** Comparison of properties among mutational classes.

		coding	CNV	cis	trans
Frequency	classified as mutant: |Z|>2.58 for YFP in haploids	7% (16/231)	10% (22/221)^1^	2% (4/231)	81% (179/221)^1^
Effects	median effect (|Z|) on YFP fluorescence in haploids^2^	48	8	8	5
	increased YFP fluorescence (Z>0)	0% (0/16)	95% (21/22)	50% (2/4)	73% (131/179)
Dominance	median effect (|Z|) on YFP fluorescence in diploids^2^	98	15	10	1
	regression coefficient (b) for YFP in haploids vs diploids	1.7	1.5	1.3	0.1
	recessive: |Z|<2.58 for YFP fluorescence in diploids	0% (0/16)	0% (0/21)	0% (0/4)	88% (151/171)
*trans* effects	median effect (|Z|) on CFP fluorescence in diploids^2^	1	4	1	1
	regression coefficient (b) for YFP vs CFP in diploids	0.00	0.37	0.08	1.04
	altered CFP fluorescence (|Z|>2.58)	13% (2/16)	81% (17/21)	25% (1/4)	65% (13/20)^3^

1 10 of the 231 mutants failed to form diploids or showed evidence of contamination and were thus not tested for changes in copy number of the reporter gene. These genotypes showed no changes in the coding sequence, promoter, or terminator of the reporter gene, thus they were either *trans*-acting or CNVs, but could not be definitively assigned.

2 Z-scores were calculated separately for YFP and CFP in haploid and diploid populations using the mean and standard deviation of control populations with the same ploidy. Therefore, the values of Z-scores should not be directly compared between haploid and diploid genotypes or between reporter genes.

3 Only the 20 *trans*-acting mutants not classified as recessive were considered.

### EMS-induced mutations affecting *P_TDH3_-YFP* are expected to be similar to spontaneous mutations affecting endogenous genes

Before discussing the evolutionary implications of our results, it is important to consider how the use of the fluorescent reporter gene *P_TDH3_-YFP* and chemical mutagen EMS might cause our data to differ from a spectrum of spontaneous mutations affecting activity of an endogenous gene.

Activity of the chimeric *P_TDH3_-YFP* reporter gene was regulated by the native *S. cerevisiae TDH3* promoter and *CYC1* terminator sequences. The *CYC1* terminator controls proper 3′ end formation of mRNA by binding to factors such as Rat1p and Sen1p that are important for the termination of many genes transcribed by DNA polymerase I and II in *S. cerevisiae* [34]. The *TDH3* gene encodes isozyme 3 of glyceraldehyde-3-phosphate dehydrogenase [35], is not required for viability under normal culture conditions [36], and is transcribed during growth on both fermentable and non-fermentable carbon sources [37] with minimal fluctuations during the cell cycle [38]. The *TDH3* promoter exemplifies regulatory principles shared by many eukaryotic genes. For example, it includes both activating [39] and repressing [37] sequences that bind transcription factors such as Gcr1p, Gcr2p, Hsf1p, Pho2p, and Rap1p [40], [41]. *TDH3* is one of the ∼19% of genes in the *S. cerevisiae* genome whose promoter has a TATA box; this is important to note because the expression of such genes appears to be more mutable than genes whose promoters lack this sequence [17]. Promoters with simple repetitive sequences have also been shown to have increased evolvability of gene expression in yeast [42], but the *TDH3* promoter appears to lack such sequences. Differences in promoter and terminator sequences among genes are expected to cause differences in gene-specific mutational spectra, but we do not expect the regulatory mutation spectrum recovered for *P_TDH3_-YFP* to be fundamentally different from that of an endogenous gene such as *TDH3*.

Unlike the regulatory sequences of *P_TDH3_-YFP*, its coding sequence was not native to yeast: it encoded a Yellow Fluorescent Protein derived from the Green Fluorescent Protein originally isolated from *Aequorea victoria* [43]. The Venus variant of YFP used in this study was previously optimized to speed maturation, improve stability, and minimize sensitivity to environmental changes such as pH and chloride concentration [18]. This optimization might explain why none of the YFP coding mutants we recovered showed increased fluorescence and suggests that mutants affecting YFP fluorescence by altering the cellular environment might be rare. Native yeast proteins might not always have such optimal activity; however, nonsynonymous mutations are generally thought to decrease a protein's function more often than they increase it, suggesting that the YFP coding sequence is not extremely unrealistic in this respect. The length and GC-content of the YFP coding region are also expected to influence the coding mutation rate measured in this study by affecting the mutational target size: at 238 amino acids, YFP is near the 20th percentile for the length of native *S. cerevisiae* proteins [44], and its GC-content of 35.56% is similar to the median GC-content for all *S. cerevisiae* genes of 39.95% (Figure S2).

The use of the chemical mutagen EMS is perhaps the most artificial element of our experimental design, although we are not the first to use it to make inferences about spontaneous mutations [45]–[48]. EMS predominantly causes G/C to A/T transitions [23], [24] and thus generates a subset of possible spontaneous mutations. However, G/C to A/T transitions are tied with G/C to T/A transversions as the most common type of spontaneous point mutation in yeast [1]. We anticipate that many types of point mutations will have similar distributions of genetic and phenotypic effects, although it will be interesting to test this hypothesis in future work. More importantly, we expect the proportion of sites targeted by EMS to be similar for coding, *cis*-regulatory, and *trans*-acting mutations, suggesting that comparing EMS-induced mutations among these classes reveals differences that should also be observed for spontaneous point mutations. We do anticipate, however, that spontaneous mutations involving more than one base-pair (e.g., insertions/deletions (indels), segmental duplications, chromosomal rearrangements) will have different distributions of effects than single point mutations, and this was observed when we compared mutants with (presumably spontaneous) duplications of *P_TDH3_-YFP* to those with single copies of the *P_TDH3_-YFP* gene. Consequently, we believe that the spectrum of mutational effects described in this work provides a reasonable approximation of the spectrum of mutational effects caused by different types of spontaneous point mutations, but might not be representative of other types of DNA lesions.

### Differences in the frequency, effects, and dominance among functional classes of mutations may impact their relative contributions to phenotypic evolution

For any mutation, the likelihood of fixation depends upon the probability that the mutational event occurs and the probability that, once it occurs, it becomes fixed within a population. This latter probability depends upon the phenotypic effects of the mutation (specifically, its impact on fitness) and (for diploid organisms) dominance. Mutations that arise more frequently have more opportunities to become fixed; mutations with larger effects on fitness should either be removed from or fixed within a population faster than mutations with smaller effects on fitness [49]; and adaptive mutations that are recessive are less likely to fix in a diploid population than equally adaptive mutations that are not recessive [50]. As described above, we observed differences in the frequency, effects (on YFP fluorescence), and dominance of different types of mutations affecting *P_TDH3_-YFP* activity. Below, we discuss how these differences might influence the evolutionary trajectories of (i) coding and regulatory mutations, (ii) *cis*-regulatory and *trans*-regulatory mutations, and (iii) copy number variants.

#### Coding and regulatory mutations

In recent years, the relative contributions of regulatory and coding changes to phenotypic evolution have been discussed extensively; however different authors have defined these categories differently [7], [15]. Some authors contrast only *cis*-regulatory and coding changes [7]–[10], [13], while others make an additional distinction between coding changes in transcription factors and coding changes in other types of proteins [11]. A broader contrast is also common in which all changes that alter gene expression are compared to changes that alter the coding sequence of the gene of interest [7], [51]. Our data are suitable for contrasting coding mutations with either *cis*-regulatory or broadly defined regulatory mutations, but we do not currently have enough information to identify which of the regulatory mutants, if any, harbor coding changes in transcription factors.

Comparing only the coding and *cis*-regulatory mutants in our collection suggests that coding mutations are more common than *cis*-regulatory mutations (at least for genes similar to *P_TDH3_-YFP*), and that *cis*-regulatory mutations have more moderate effects (Table 1). Both classes of mutations showed similar effects in haploid and diploid cells, suggesting that they are both non-recessive and selectable as soon as they arise in diploid populations. If the large changes in *P_TDH3_-YFP* activity we observed in the majority of coding mutations tend to be strongly deleterious, the more moderate effects of *cis*-regulatory mutations (especially when coupled with the presumed lower degree of pleiotropy of *cis*-regulatory mutations) might make them more likely to fix than coding mutations despite their lower mutation rate. This might be especially true when a favored phenotype requires an increase in gene activity, as this type of change was observed for 50% of the *cis*-regulatory mutants recovered, but none of the coding mutants.

The broader definition of “regulatory” mutants described above includes all genotypes classified as *cis*-regulatory, *trans*-acting, and copy number mutants in this study because they all change YFP fluorescence without altering the coding sequence of YFP. Considering these three classes together, regulatory mutations were over 10-times more common than coding mutations in *P_TDH3_-YFP*. And this is without accounting for the fact that the gene duplication rate was not expected to be increased by EMS. Regulatory mutations (broadly defined) may therefore be a much more abundant source of variation in a gene's activity than mutations changing its protein sequence. This higher frequency might be at least partially offset, however, by the fact that over 75% of the regulatory mutants we characterized (all of which were categorized as *trans*-acting) appeared to be recessive. Like *cis*-regulatory mutants, this broader class of regulatory mutants had more moderate effects on YFP fluorescence than coding mutants and the activity of *P_TDH3_-YFP* was increased at least half of the time. The frequent recovery of regulatory mutants with elevated YFP fluorescence was unexpected and particularly surprising given that the *TDH3* promoter drives high levels of expression in wildtype cells [52] and Tdh3p is in the 98th percentile for protein abundance in *S. cerevisiae* [53]. It will be interesting to see in future work whether mutational spectra for other genes also show a high rate of mutations that increase the gene's activity.

#### *cis*-regulatory and *trans*-acting changes

The relative contribution of *cis*- and *trans*-acting mutations to polymorphic and divergent gene expression has also been examined in a variety of species, either using genetic mapping or allele-specific expression to infer changes in *cis*- and *trans*-regulation (reviewed by [14], [54]–[56]). These analyses have revealed differences in the frequency, effects, and dominance of segregating *cis*- and *trans*-regulatory variation that our data suggest might result (at least in part) from inherent properties of *cis*- and *trans*-regulatory mutations rather than selection for a biased subset of regulatory mutations.

For example, *trans*-regulatory variation has been observed to be more abundant within a species than *cis*-acting variation, and we found that the mutational target size for *trans*-acting mutations affecting activity of *P_TDH3_-YFP* was ∼45 times larger than for *cis*-regulatory mutations affecting activity of this gene. A larger mutational target size for *trans*-regulatory variation was also inferred from mutation accumulation lines [17], [57]. *cis*-acting quantitative trait loci affecting gene expression (eQTL) generally have larger average effects on a gene's expression than *trans*-regulatory eQTL, and we found that *cis*-regulatory mutations trended toward having larger effects on YFP fluorescence than *trans*-regulatory mutations in both haploid (P = 0.07, one-sided MWW test) and heterozygous diploid (P = 0.0007, one-sided MWW test) cells. Finally, segregating *cis*-regulatory alleles have been shown to be recessive less often than *trans*-regulatory alleles [58], [59], and we found that the effects of *cis*-regulatory mutants in heterozygous cells were masked less often than the effects of *trans*-acting mutants.

Taken together, these observations suggest that, *trans*-acting variation might be more common within a species than *cis*-regulatory variation for individual genes because of the higher rate of *trans*-acting mutations, their tendency to have smaller effects on gene activity than *cis*-acting mutations, and their propensity to be recessive in diploid cells. If the size of the effect on gene activity is correlated with the selection coefficient, *cis*-regulatory variants might contribute more to expression differences between than within species (as has been observed for *Drosophila* [60] and *Saccharomyces* [61]) because the tendency of *cis*-regulatory mutations to have larger and more non-recessive effects on gene activity than *trans*-regulatory mutations should cause them to be selected for or against more strongly. This should lower the probability that a *cis*-regulatory mutation segregates for long periods of time within a species and raise the probability that a *cis*-regulatory mutation contributes to adaptive regulatory changes between species.

#### Copy number variants

Gene duplications or deletions can alter the number of copies of a gene and, assuming all copies are expressed, this will affect the abundance of the gene's product. Such copy number variants are known to contribute to phenotypic diversity in humans, yeast and other eukaryotes [62]–[64]. In *S. cerevisiae*, spontaneous gene duplications are almost 5 times more common than spontaneous point mutations: in one genome replication, 0.019 gene duplications are expected, compared to only 0.004 point mutations [1]. Consistent with this high spontaneous mutation rate, duplications containing *P_TDH3_-YFP* were found in 10% of the mutants we examined despite the fact that EMS is not expected to influence their occurrence. Duplications of other genomic regions that affect *P_TDH3_-YFP* activity might also be present in our mutant collection as *trans*-acting mutants, but determining the frequency and location of any such duplications (as well as the extent of the duplications including *P_TDH3_-YFP*) requires further analysis. As expected, nearly all mutant genotypes harboring a duplication of *P_TDH3_-YFP* increased the YFP fluorescence of both haploid and diploid cells. The one exception was a mutant that showed decreased YFP fluorescence, which could be caused by the duplication of one or more negative regulators of *P_TDH3_-YFP*. Interestingly, the majority of duplications that increased YFP fluorescence decreased CFP fluorescence in diploids, which might reflect a mechanism that silences both the original and new copy of a gene following a duplication event [65]. Despite their high mutation rate, copy number variants appear to rarely be fixed between yeast species [66], suggesting that they are often deleterious and eliminated by natural selection or have a high rate of spontaneous reversion.

### Unresolved questions: Pleiotropy, fitness, and the genomic locations of mutations affecting *P_TDH3_-YFP* activity

This study provides an unprecedented survey of the functional characteristics of new mutations; however, our understanding of mutational properties remains far from complete, even for the *P_TDH3_-YFP* reporter gene. For example, the effect of a mutation on fitness is what matters most for evolution, but we measured only the effects of mutations on YFP fluorescence. If fluorescence were an adaptive trait, a relationship between mutational effects on *P_TDH3_-YFP* activity and fitness is expected, but the effects of each mutation on other traits (i.e., pleiotropy) will also influence fitness, complicating this relationship. Similarly, we assessed the dominance of mutant effects on YFP fluorescence, but dominance at the level of a single gene's activity might not always translate to dominance at the level of higher-order phenotypes, which are more likely to be the targets of natural selection.

The unique collection of mutants described here provides a rare opportunity to address these issues, however, by mapping each of the mutations affecting *P_TDH3_-YFP* activity, engineering them individually into the ancestral genetic background, and directly measuring pleiotropy (quantified as the number of genes in the genome that change expression in response to the mutation) and fitness under different conditions (quantified as the effect of the mutation on relative growth rate). Determining the identity of mutations responsible for these mutant phenotypes will also allow us to assess their distribution within the genome and among factors expected to influence *P_TDH3_-YFP* activity and to compare the overall contributions of coding and non-coding changes to the mutational spectrum for *P_TDH3_-YFP*. Integrating these data with those presented here, as well as performing similar analyses of reporter genes using promoters from other *S. cerevisiae* genes, should greatly improve our ability to predict the types of genetic changes most likely to contribute to phenotypic evolution under different conditions.

## Materials and Methods

An abbreviated version of the materials and methods follows. Complete materials and methods, including calculation of the spontaneous mutation rate, are included as Supporting Information (Text S1).

### Mutagenesis

Chemical mutagenesis with Ethyl Methane Sulfonate (EMS) was performed as previously described [67], except that the volume of the cell suspension was doubled to 2 ml, the cell density was reduced by 50% to 6×10^7^ cells/ml, the concentration of EMS was reduced by 75% to 7.5%, and the time of exposure was reduced by 25% to 45 minutes. Following mutagenesis, control and mutagen-treated cells were cultured at 30–32°C for 42 hours in arginine dropout liquid media (Synthetic Complete media lacking arginine) [67].

### Canavanine resistance assay

The canavanine resistance mutation rate in the EMS-treated population was calculated by comparing colony forming units on arginine dropout plates with and without 60 mg/l canavanine sulfate (Sigma-Aldrich, St. Louis, MO) [67].

### Flow cytometry and primary screen for prospective mutants

Prior to analysis and sorting, cells from the control culture were stained with Cy5 (GE Healthcare, Piscataway, NJ) so that they could be distinguished from EMS-treated cells when analyzed simultaneously. Aliquots of both populations were mixed together in Phosphate Buffered Saline (PBS) for analysis and sorted in a FACSaria flow cytometer/cell sorter (BD Biosystems, San Jose, CA). The sorting and analysis of the mixed suspensions was restricted to a FSC-defined subset of events to reduce the influence of non-cell particles. In each of nine consecutive sorting runs, 96 events were collected from each tail of the EMS-treated and control populations, for a total of 384 events collected in each sorting run. Thresholds used for sorting during each run are presented in Table S1. Sorted events were arrayed onto YPD agar plates [67], then incubated at 30°C for 28 hours.

### Liquid cultures for secondary screen of candidate mutants and diploid testing

High-throughput parallel liquid culturing of genotypes was performed by inoculating from a colony or patch and culturing for 24 hours at 30°C in 96-well deep well plates in YPD. Saturated cultures were diluted 100× into arginine dropout liquid and cultured at 30°C for at least 2 doublings until the density reached 0.5–1.0×10^7^ cells/ml.

### Quantification of fluorescence in flow cytometry data

Haploid YFP fluorescence and FSC were evaluated by a C6 flow cytometer (Accuri, Ann Arbor, MI); diploid YFP, CFP, and FSC were evaluated by a FACSaria flow cytometer (BD Biosciences, San Jose, CA). After log-transformation of FSC and fluorescence values, filters were applied to cull events with extreme FSC values; generally, these filters corresponded to approximately the 20th and 80th percentiles of FSC. The fluorescence phenotype of each genotype was defined as its median YFP/FSC or CFP/FSC ratio. This ratio was converted to a Z-score using the mean and standard deviation calculated from at least 10 (and up to 143) replicate control cultures containing cells with the unmutagenized, ancestral genotype.

### Pyrosequencing to assay YFP copy number

PCR primers hybridizing to DNA sequences shared by YFP and CFP that flanked a position of dissimilarity were used to amplify a small region of DNA for analysis. Using the PSQ96 pyrosequencer (Qiagen, Valencia, CA), an internal sequencing primer was hybridized to these amplified fragments and extended to a diagnostic position that differed between YFP and CFP, allowing the relative frequency of YFP and CFP alleles to be compared in heterozygous diploid cells.

### Testing the sufficiency of promoter mutations

After introducing each promoter mutation into the unmutagenized ancestral genotype using site-directed mutagenesis, we quantified YFP fluorescence using the C6 flow cytometer (Accuri, Ann Arbor, MI). Two 80,000-event samples were collected for genotypes into which the mutations identified at positions −255, −240, and −140 had been introduced into the unmutagenized progenitor, a strain in which a wildtype copy of the promoter had been re-introduced in parallel, and the four mutant genotypes in which the promoter mutations were originally detected (including the two different isolates with the mutation at −255). Taking the median YFP/FSC as the fluorescence phenotype of each culture, we compared the genotypes with site-directed promoter mutations to the reengineered wildtype control, and the genotypes with site-directed promoter mutations to the mutant(s) in which it was originally observed using MWW tests.

## Supporting Information

Figure S1 A minority of mutant genotypes showed increased variance in YFP fluorescence. The histograms of Z-scores shown represent the variance in YFP fluorescence for genotypes isolated from the control (black) and EMS-treated populations. EMS-treated genotypes with (green) and without (red) a statistically significant change in median YFP fluorescence relative to the control population are plotted separately, but the frequency of each class was calculated using the total number of EMS-treated genotypes tested. These Z-scores were calculated as described for median YFP fluorescence: the difference between variance in YFP fluorescence for a particular genotype and the mean variance of all control genotypes was divided by the standard deviation of variance values among the control genotypes. Of the 231 EMS-treated genotypes classified as mutants for YFP fluorescence (green), 47 (11 CNV, 5 coding, and 26 *trans-*acting mutants) showed a significant (|Z|>2.58) change in variance. By comparison, 37 of the 833 EMS-treated genotypes without a significant change in median YFP-fluorescence (red) and 20 of the 1137 genotypes from the control population (black) showed statistically significant changes in variance.
(PDF)

Figure S2 The GC content of YFP is similar to that of native *S. cerevisiae* genes. Because EMS preferentially causes mutations at G-C base pairs, we compared the percentage of nucleotides that are either guanine or cytosine (%GC) in the YFP coding sequence to the %GC in coding sequences of genes endogenous to the *S. cerevisiae* genome. A histogram summarizing the %GC for all genes in the annotated *S. cerevisiae* genome (median %GC = 39.95%) is shown with the %GC of YFP (35.56%) indicated with a red arrow. Sequences for *S. cerevisiae* genes are from the Saccharomyces Genome Database EF3 assembly (Ensembl, Release 64).
(PDF)

Table S1 Percentile thresholds used for sorting in replicate populations.
(PDF)

Table S2 YFP fluorescence for all 2201 genotypes recovered from fluorescence activated cell sorting (FACS).
(XLS)

Table S3 Assignment to mutant class and effects on fluorescence phenotypes in haploid and diploid cells for all 231 genotypes considered mutants.
(XLS)

Table S4 Estimation of spontaneous mutation rate for *P_TDH3_-YFP* activity based on validated mutants.
(PDF)

Text S1 Expanded Materials and Methods.
(PDF)
