# Supplementary material for: Contrasting Properties of Gene-Specific Regulatory, Coding, and Copy Number Mutations in Saccharomyces cerevisiae: Frequency, Effects, and Dominance
Source: PLoS Genet. 2012 Feb 9;8(2):e1002497. doi: 10.1371/journal.pgen.1002497 (PMC3276545; doi:10.1371/journal.pgen.1002497)
Supplement: Figure S1 — A minority of mutant genotypes showed increased variance in YFP fluorescence. The histograms of Z-scores shown represent the variance in YFP fluorescence for genotypes isolated from the control (black) and EMS-treated populations. EMS-treated genotypes with (green) and without (red) a statistically significant change in median YFP fluorescence relative to the control population are plotted separately, but the frequency of each class was calculated using the total number of EMS-treated genotypes tested. These Z-scores were calculated as described for median YFP fluorescence: the difference between variance in YFP fluorescence for a particular genotype and the mean variance of all control genotypes was divided by the standard deviation of variance values among the control genotypes. Of the 231 EMS-treated genotypes classified as mutants for YFP fluorescence (green), 47 (11 CNV, 5 coding, and 26 trans-acting mutants) showed a significant (|Z|>2.58) change in variance. By comparison, 37 of the 833 EMS-treated genotypes without a significant change in median YFP-fluorescence (red) and 20 of the 1137 genotypes from the control population (black) showed statistically significant changes in variance. (PDF) [file pgen.1002497.s001.pdf]

Gruber et al.
Figure S1

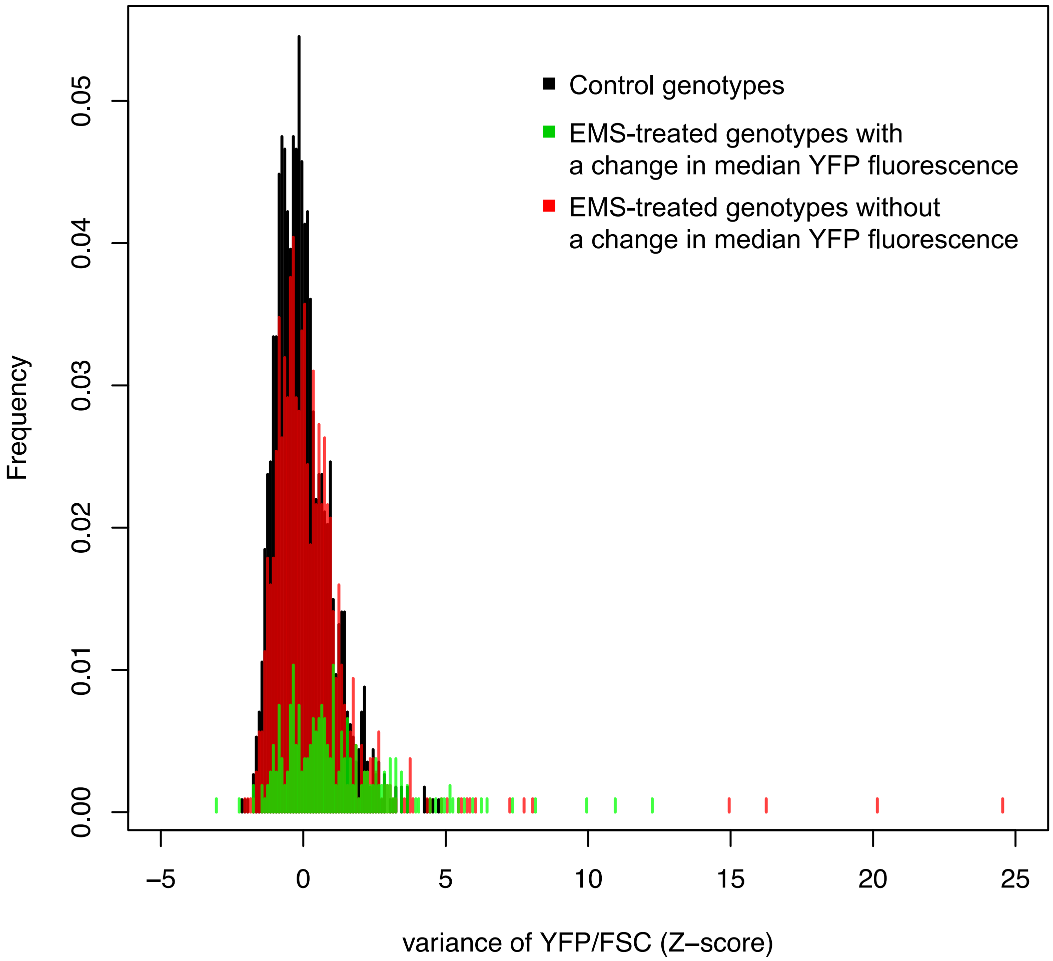

A minority of mutant genotypes showed increased variance in YFP fluorescence. The histograms of Z-scores shown represent the variance in YFP fluorescence for genotypes isolated from the control (black) and EMS-treated populations. EMS-treated genotypes with (green) and without (red) a statistically significant change in median YFP fluorescence relative to the control population are plotted separately, but the frequency of each class was calculated using the total number of EMS-treated genotypes tested. These Z-scores were calculated as described for median YFP fluorescence: the difference between variance in YFP fluorescence for a particular genotype and the mean variance of all control genotypes was divided by the standard deviation of variance values among the control genotypes. Of the 231 EMS-treated genotypes classified as mutants for YFP fluorescence (green), 47 (11 CNV, 5 coding, and 26 trans-acting mutants) showed a significant ($|Z| > 2.58$) change in variance. By comparison, 37 of the 833 EMS-treated genotypes without a significant change in median YFP-fluorescence (red) and 20 of the 1137 genotypes from the control population (black) showed statistically significant changes in variance.
