# Supplementary material for: Contrasting Properties of Gene-Specific Regulatory, Coding, and Copy Number Mutations in Saccharomyces cerevisiae: Frequency, Effects, and Dominance
Source: PLoS Genet. 2012 Feb 9;8(2):e1002497. doi: 10.1371/journal.pgen.1002497 (PMC3276545; doi:10.1371/journal.pgen.1002497)
Supplement: Figure S2 — The GC content of YFP is similar to that of native S. cerevisiae genes. Because EMS preferentially causes mutations at G-C base pairs, we compared the percentage of nucleotides that are either guanine or cytosine (%GC) in the YFP coding sequence to the %GC in coding sequences of genes endogenous to the S. cerevisiae genome. A histogram summarizing the %GC for all genes in the annotated S. cerevisiae genome (median %GC = 39.95%) is shown with the %GC of YFP (35.56%) indicated with a red arrow. Sequences for S. cerevisiae genes are from the Saccharomyces Genome Database EF3 assembly (Ensembl, Release 64). (PDF) [file pgen.1002497.s002.pdf]

Gruber et al.
Figure S2

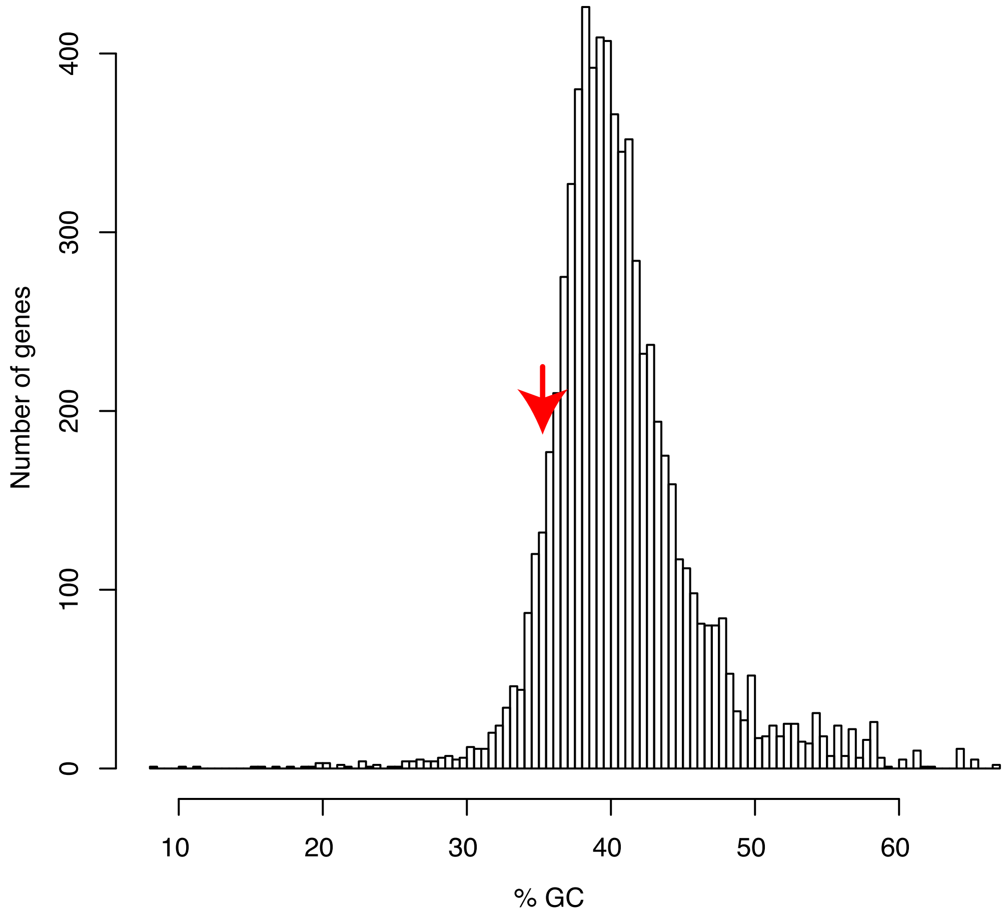

The GC content of YFP is similar to that of native *S. cerevisiae* genes. Because EMS preferentially causes mutations at G-C base pairs, we compared the percentage of nucleotides that are either guanine or cytosine (%GC) in the YFP coding sequence to the %GC in coding sequences of genes endogenous to the *S. cerevisiae* genome. A histogram summarizing the %GC for all genes in the annotated *S. cerevisiae* genome (median %GC = 39.95%) is shown with the %GC of YFP (35.56%) indicated with a red arrow. Sequences for *S. cerevisiae* genes are from the Saccharomyces Genome Database EF3 assembly (Ensembl, Release 64).
