# Supplementary material for: Contrasting Properties of Gene-Specific Regulatory, Coding, and Copy Number Mutations in Saccharomyces cerevisiae: Frequency, Effects, and Dominance
Source: PLoS Genet. 2012 Feb 9;8(2):e1002497. doi: 10.1371/journal.pgen.1002497 (PMC3276545; doi:10.1371/journal.pgen.1002497)
Supplement: Table S1 — Percentile thresholds used for sorting in replicate populations. (PDF) [file pgen.1002497.s003.pdf]

Table S1. Percentage of each subpopulation more extreme than sorting threshold

	**Low fluorescence**		**High Fluorescence**	
run ID	*Control*	*EMS*	*Control*	*EMS*
1	0.47	0.79	0.99	1.49
2	0.99	1.39	0.56	0.96
3	1.00	1.38	0.56	0.91
4	1.05	1.40	0.51	0.89
6	0.86	1.24	0.55	0.88
7	0.89	1.35	0.57	0.95
8	0.76	1.17	0.67	1.09
9	0.68	1.10	0.68	1.11
10	0.72	1.14	0.67	1.12
Mean	0.82	1.22	0.64	1.04
