# Supplementary material for: Contrasting Properties of Gene-Specific Regulatory, Coding, and Copy Number Mutations in Saccharomyces cerevisiae: Frequency, Effects, and Dominance
Source: PLoS Genet. 2012 Feb 9;8(2):e1002497. doi: 10.1371/journal.pgen.1002497 (PMC3276545; doi:10.1371/journal.pgen.1002497)
Supplement: Table S4 — Estimation of spontaneous mutation rate for PTDH3-YFP activity based on validated mutants. (PDF) [file pgen.1002497.s006.pdf]

Table S4: Estimation of P_{TDH3}-*YFP* fluorescence mutation rate from validated mutants

All validated mutants

Phenotype	cell fraction[1]	relative viability[2]	c.f.u. fraction[3]	mutants[4]	screened[5]	frequency[6]	mutant frequency[7]
High YFP	0.010	0.784	0.008	162	503	0.322	0.0026
Typical YFP	0.977	1.000	0.977				
Low YFP	0.012	0.843	0.010	69	561	0.119	0.0012
Total	1.000		0.996	231	1064		0.0039
						[8]Inflaction factor due to EMS	5797
						**[9]Spontaneous mutation rate (x10^{-7})**	6.6476

Validated mutants, excluding known CNVs

Phenotype	cell fraction[1]	relative viability[2]	c.f.u. fraction[3]	mutants[4]	screened[5]	frequency[6]	mutant frequency[7]
High YFP	0.010	0.784	0.008	141	503	0.280	0.0023
Typical YFP	0.977	1.000	0.977				
Low YFP	0.012	0.843	0.010	68	561	0.119	0.0012
Total	1.000		0.996	209	1064		0.0035
						[8]Inflaction factor due to EMS	5797
						**[9]Spontaneous mutation rate (x10^{-7})**	6.0605

[1] proportion of all FACS events ("cells") with this phenotype
[2] proportion of c.f.u.with this phenotype relative to the proportion of c.f.u. in the wild-type YFP expression class
[3] proportion of all c.f.u. with this phenotype
[4] number of c.f.u. with signicant changes in YFP fluorescence
[5] number of c.f.u. subjected to secondary screening
[6] proportion of c.f.u. screened that are fluorescence mutants
[7] frequency of fluorescence mutants among all c.f.u. in the population used for sorting
[8] measured by comparing the observed frequency of canavanine resistance mutants to a published estimate of the spontaneous canavanine resistance mutation rate.
[9] spontaneous mutation rate of fluorescence changes suggested by observed EMS-induced mutant frequency; measured per haploid genome, per generation.
