# Supplementary material for: Contrasting Properties of Gene-Specific Regulatory, Coding, and Copy Number Mutations in Saccharomyces cerevisiae: Frequency, Effects, and Dominance
Source: PLoS Genet. 2012 Feb 9;8(2):e1002497. doi: 10.1371/journal.pgen.1002497 (PMC3276545; doi:10.1371/journal.pgen.1002497)
Supplement: Text S1 — Expanded Materials and Methods. (PDF) [file pgen.1002497.s007.pdf]

Text S1: Expanded Materials and Methods

Yeast Strains

The CFP and YFP expressing strains were derived from BY4722 (**MAT**α, *leu2Δ0*, *ura3Δ0*, *CAN1*^{S}) and BY4724 (**MAT**a, *lys2Δ0*, *ura3Δ0*, *CAN1*^{S}), respectively, and are descended from the S288C background [69]. BY4722 and BY4724 were initially engineered by inserting a chimeric P_{TDH3}-*GFP-CYC1terminator* gene between positions 199270 and 199271 of Chromosome 1. This gene consisted of a copy of the complete *TDH3* 5' intergenic sequence (the reverse complement of NC_001139.9:883811-884488, except for a T->C polymorphism at position 884103), the yeast Enhanced Green Fluorescent Protein (yEGFP) [70], and the *CYC1* "terminator" 3'UTR [19]; yEGFP strains were constructed and provided by B. Williams (Michigan State University), and resequenced by the authors of this work. To generate a pair of reporter genes whose individual expression could be distinguished, the BY4724 yEGFP sequence was altered to encode the venus variant of Yellow Fluorescent Protein (YFP) [18], and the BY4722 yEGFP sequence was changed to the cerulean Cyan Fluorescent Protein (CFP) [25]. The YFP and CFP coding sequences differ by 22 of 714 nucleotides.

Transformations

Transformations were performed according to the LiAc/PEG method of high-efficiency transformation [71]. To alter specific nucleotides, the *URA3* gene was targeted to replace the region of interest, and transformants were selected on Synthetic Complete media lacking uracil [67]. The desired new sequence was targeted to replace this *URA3* region, and transformants were selected on 5-Fluoro-orotic acid (5FOA) plates [67]. Successful transformation was verified by PCR and sequencing of the modified region.

Mutagenesis

Chemical mutagenesis with Ethyl Methane Sulfonate (EMS) was performed on a clonal population of YFP-expressing cells following standard protocols [67], except that the volume of cell suspension was doubled to 2 ml, the cell density was reduced by 50% to 6 x 10^7 cells/ml, the concentration of EMS was reduced by 75% to 7.5%, and the time of exposure was reduced by 25% to 45 minutes. Following mutagenesis, control and mutagen-treated cells were cultured at 30-32°C for 42 hours in arginine dropout liquid media (Synthetic Complete media lacking arginine) [67]. During this period, dilutions were performed at 19, 28, and 38 hours to support continuous exponential growth, maintaining a minimum population size of 10^6 cells. The starting density resulting from each dilution was matched between mutagen-treated and control cultures. Serial dilutions from the final culture on arginine dropout plates indicated a final density of ~4 x 10^7 colony forming units (cfu) per ml in both final cultures.

Canavanine resistance assay

We estimated the canavanine resistance mutation rate as a measure of the *de facto* strength of the mutagenesis treatment. Using serial dilutions, the canavanine resistance mutation rate in the EMS-treated population was calculated by comparing cfu on arginine dropout plates with and without 60 mg/l canavanine sulfate (Sigma-Aldrich, St. Louis, MO) [67]. An inflation factor was calculated by dividing this rate by a published estimate [22] of the spontaneous mutation rate.

Estimating the frequency of EMS-induced mutations per bp and genome-wide

Mutagenesis conditions were optimized to maximize recovery of genotypes with a single mutation that affects P_{TDH3}-*YFP* activity; however, each genotype was also expected to harbor

other mutations in its genome that do not affect P_{TDH3}-*YFP* activity. To estimate the expected number of total mutations per genome, we considered the types of mutations induced by EMS as well as the difference between the observed EMS-induced and spontaneous canavanine resistance mutation rates. The overwhelming majority of mutations caused by EMS are G-->A (or C-->T) transitions [24]; indeed, all of the mutations in P_{TDH3}-*YFP* we identified (other than duplications) were of this type (Table S3). We took this bias into account when extrapolating from the observed canavanine resistance mutation rate by considering only the subset of the genome, and the subset of the canavanine susceptibility gene (*CAN1*), expected to be mutated by EMS (*i.e.*, the G/C basepairs).

We first estimated the number of G/C positions in *CAN1* that could lead to the canavanine resistance phenotype if mutated to A/T. By mutating all possible *CAN1* G/C positions to A/T *in silico*, we noted that there are 44 possible G/C basepairs that, when mutated to A/T, result in stop codons. However, we assumed that the mutational target for canavanine resistance would include these 44 positions as well as an unknown number of G/C --> A/T missense (*i.e.*, amino acid changing) mutations; an unknown fraction of all possible missense mutations will disrupt *CAN1* function enough to confer canavanine resistance. To estimate this number, we followed the logic of [22], which reported the sequence basis of 227 spontaneous canavanine resistant mutants (Figure 3 in [22]). Of these, 20 are nonsense (*i.e.*, causing a stop codon) mutations due to a change of G/C --> A/T. Thus the recovery rate of G/C --> A/T nonsense mutations in [22] was estimated as 20/44. Following their reasoning, we made the assumption that the recovery rate for canavanine-resistance mutations was the same for both nonsense and missense mutations. In their dataset, we counted 20 G/C --> A/T missense mutations. Therefore, if the estimate of the recovery rate was 20/44, and there were 20 observed G/C --> A/T missense mutations, we estimated that there are 44 possible G/C --> A/T missense mutations. Therefore,

we estimated that there are 88 G/C --> A/T mutations (44 nonsense and 44 missense) that, when induced by EMS, will lead to canavanine resistance.

We used this estimate of the [EMS-specific] mutational target at *CAN1* to convert our phenotypic canavanine resistance rate to a per basepair --G/C only-- mutation rate, which in turn we used to estimate the number of mutations per genome. Our observed phenotypic mutation rate for canavanine resistance was 8.72 mutants / 10,000 cells. Given the estimate of 88 bp in each cell in which such a mutation can occur, it implies that there were 8.72 mutations / (88 bp/genome * 10,000 genomes) = 9.91×10^{-6} mutations per G/C basepair. Assuming that only G/C positions are mutable, to convert the per-basepair mutation rate to the average number of mutations per genome, we calculated that the 16 *S. cerevisiae* chromosomes contain 4.622 Mbp G/C (http://www.yeastgenome.org/composition.html). Assuming that the per-basepair mutation rate at *CAN1* is representative of the whole genome, we calculated that (9.91×10^{-6} mutations/bp) * (4.622×10^{6} bp/genome) = 45.8 mutations per genome. Assuming that the number of mutations per genome follows a Poisson distribution with a mean of 45.8, 99% of cells recovered from our screen are expected to contain 30 - 64 nucleotide changes per genome.

Flow cytometry and primary screen for prospective mutants

Prior to analysis and sorting, cells from the control culture were stained so that they could be distinguished from EMS-treated cells when analyzed simultaneously. Cells from liquid culture were washed with cold Phosphate Buffered Saline (PBS). After removing PBS, the pellet was resuspended in 250 μl Cy5 Post-labeling Reactive Dye (GE Healthcare, Piscataway, NJ) in PBS and placed on ice for 10 minutes, washed once and resuspended in cold PBS. The EMS-treated and control cultures were processed identically except for the addition of pure PBS to the

former, but Cy5 dye to the latter. Aliquots of both populations were mixed together in PBS for analysis by Fluorescence Activated Cell Sorting (FACS). To determine whether Cy5 staining affected the measured fluorescence of the YFP reporter gene, we mixed Cy5-labeled and unlabeled cells from the same control culture of P_{TDH3}-*YFP*. No significant difference in YFP fluorescence was observed between the labeled and unlabeled subpopulations (*P*=0.9, t-test) in the size interval used for sorting analysis.

To characterize the effects of mutagen on YFP fluorescence and create a collection of prospective mutants, we analyzed control and mutagenized cells in a FACSaria flow cytometer/cell sorter (BD Biosystems, San Jose, CA). For each cell, FACS measured the fluorescence of Cy5 and YFP and provided an estimate of cell size using measurements of "Forward Scatter" (FSC). In clonal populations, fluorescence of "events" (cells and other fluorescent particles) showed a linear correlation with FSC (Figure 1A). The sorting and analysis of the mixed suspensions was restricted to an FSC-defined subset of events to reduce the influence of non-cell particles. In each of nine consecutive sorting runs, 192 events each were collected from the EMS-treated and control populations (as defined by absence or presence of Cy5 fluorescence, respectively). These 192 events per population were evenly divided between those that showed low levels of YFP fluorescence (within or less than approximately the least-fluorescent percentile of the control population) and high levels of YFP fluorescence (within or greater than approximately the most-fluorescent percentile of the control population). The precise cut-offs used in each run are presented in Table S1. These events were arrayed by the FACS machine onto solid YPD plates [67] in a 384-position grid and incubated at 30°C for 28 hours. 1% of the 3456 sorted events resulted in two colonies, indicating that two different cells had been deposited in the same grid position. These cases were excluded from further analysis.

Estimation of P_{TDH3}-*YFP* fluorescence mutation rate from EMS-treated population

FSC, YFP, and Cy5 fluorescence recorded during FACS sorting runs were log-transformed. The FSC-defined subpopulation described above contained approximately 600,000 events per run. Within this range, the ratio YFP/FSC of each cell was defined as its YFP fluorescence phenotype. To estimate the mutation rate for mutations affecting YFP fluorescence, we reasoned that increases in the number of cells with extreme YFP fluorescent phenotypes in the EMS-treated population represented the input of additional genetic variation in the form of the G/C-to-A/T point mutations characteristic of EMS treatment. (In contrast to these point mutations, spontaneous Copy Number Variants are not expected to be induced by EMS, so their frequency should be similar in the EMS-treated and control populations.) To compare the distribution of fluorescence phenotypes between EMS-treated and control populations, we producing an "enrichment curve" (Figure 1C) by assigning the phenotypes of each population's cells into ~500 bins and comparing the frequency of cells in each bin between populations. High and low fluorescence areas of enrichment were summed to estimate the frequency (f) of EMS-induced mutants with altered activity of P_{TDH3}-*YFP*; and the remainder in the midrange ($1-f$) estimated the fraction of the EMS-treated subpopulation with zero relevant mutations (P_0). Assuming that relevant mutations are Poisson distributed among genomes, we calculated the average rate of 0.0303 mutations per genome. Given this mutation rate, 2.94% of cells are expected to have exactly one such mutation, whereas only 0.04% of cells are expected to have more than one such mutation. To estimate the spontaneous mutation rate, the EMS-induced mutation rate was divided by the observed mutation rate inflation factor (from canavanine resistance).

Liquid cultures for secondary screen of candidate mutants and diploid testing

High-throughput parallel liquid culturing of genotypes was implemented so that the fluorescence phenotypes of many representative cells of a given genotype could be compared to those of the other genotypes. Liquid cultures were inoculated from a colony or patch and cultured for 24 hours at 30°C in 96-well deep well plates in YPD. Saturated cultures were diluted 100X into arginine dropout liquid and cultured at 30°C for at least 2 doublings until the density reached 0.5 - 1.0 x 10^7 cells/ml. For the secondary screen (i.e., haploids), each of the 384 genotypes isolated on the same plate during the primary screen were cultured simultaneously as a block.

Statistical identification of mutants with altered *P_{TDH3}-YFP* activity

YFP fluorescence and FSC of cells in a clonal culture derived from each isolate were evaluated in a C6 flow cytometer (Accuri, Ann Arbor, MI) fed by a Hypercyt Autosampler (Intellicyt, Albuquerque, NM) as a secondary screen. Populations with fewer than 5000 events were excluded from further analysis. After log-transformation of FSC and YFP fluorescence values, filters were applied to cull events with extreme FSC values; on average, these filters culled approximately the smallest 16% and largest 23% of cells. Within this central range of FSC values, a linear correlation between YFP and FSC was observed (Figure 1A) and the fluorescence phenotype of each candidate mutant was defined as its median YFP/FSC ratio.

The median value of YFP fluorescence per unit FSC from each candidate mutant genotype was compared to a block-specific null distribution composed of median fluorescence values from replicate control cultures. These cultures were descended from isolates collected from the control subpopulation on the same 384-well plate. The phenotype of every sample was converted to a Z-score, calculated as the deviation from the mean of the corresponding null distribution divided by the standard deviation of the corresponding null distribution. Genotypes

with an absolute value of this score ≥ 2.58 (i.e., a median outside of the 99% confidence interval of the block-specific null distribution) were considered mutants. All analyses were conducted in the statistical programming language R [72].

Alternative estimation of spontaneous mutation rate

The spontaneous mutation rate for P_{TDH3}-*YFP* activity can also be estimated using the proportion of sorted cells classified as mutants following the secondary screen (Table S4). This calculation takes into account the frequency of colony-forming FACS events classified as having high, low, or typical YFP fluorescence (as defined by the thresholds listed in Table S1) as well as the frequency of genotypes with a significant change in median YFP fluorescence (*i.e.*, mutants) among the cells sorted from the high and low fluorescence tails of the distribution. Including CNVs resulted in an estimated spontaneous mutation rate of 6.65 x 10^{-7} per haploid genome per generation, whereas excluding them (because they are unlikely to have resulted from the EMS mutagenesis treatment) resulted in an estimated mutation rate of 6.06 x 10^{-7} per haploid genome per generation. Note that these estimates are an order of magnitude lower than the spontaneous mutation rate we estimated by comparing the full EMS-treated and control populations, suggesting that many mutations had effects on P_{TDH3}-*YFP* activity that were too small for our secondary screen to detect.

Sequencing

We sequenced the transgenic reporter gene with a 1821 bp amplicon (PCR primers Forward 5'-gcatttttaccaagcagtcgtc-3' and Reverse 5'-gctgtcccaaagccacttc-3') from each verified mutant genotype. 143 bp flanking the 1678 bp gene were included to ensure complete high-quality sequence of the inserted transgene. For template, colony picks were boiled in 10 μl NaOH (0.02

M) for 5 minutes (http://openwetware.org/wiki/Blackburn:Yeast_Colony_PCR_v2.0). The PCR cocktail contained 1M Betaine (Sigma-Aldrich, St. Louis, MO) but was otherwise standard (conditions available upon request). The amplicon was sequenced with three primers ((1) the Forward PCR primer, (2) 5'-ggttgaaaccagttccctga-3', and (3) 5'-caagaccagagctgaagtcaag-3') by the University of Michigan Sequencing Core. Given the G/C-specificity of EMS [24], the estimated per-bp mutation rate (9.91 x 10^{-6} mutations/bp), and a Poisson distribution of mutations, we calculated that 99% of the time a random region of DNA with 601 GC bases (the number contained in the P_{TDH3}-*YFP gene)* is sequenced in 231 genotypes, four or fewer total mutations should be observed among all strains.

Mating mutants into diploid background

In preparation for evaluation of fluorescence in diploids, 30 mutants and 10 representatives of the ancestral YFP genotype were patch cultured in random order on YPD plates. This structure (40-sample blocks) was preserved throughout mating, liquid culture, flow cytometry, and analysis. The CFP strain was cultured as a lawn on YPD plates. After mating, diploid patches were selected by replica plating onto selective plates lacking leucine and lysine (mated diploids, but neither of the haploids, were prototrophic for both lysine and leucine).

Quantification of YFP and CFP fluorescence phenotypes in diploids

From approximately 20,000 events per diploid sample, FSC and YFP fluorescence were recorded by the FACSaria as described above; CFP fluorescence was also recorded. The FSC, YFP, and CFP values of each recorded event were log-transformed. An FSC (cell size) filter was applied that culled the smallest (~18%) and largest (~20%) cells of each sample. Furthermore, events not showing evidence of fluorescence from both YFP and CFP were

excluded as probable haploid contaminants, except in the few samples for which no events showed YFP fluorescence (as expected for mutations causing severe reduction in YFP expression or function). Samples for which >9,000 events remained after filtering were considered. The sample's fluorescence phenotypes were defined as the median YFP/FSC and CFP/FSC values. As with the haploid data, a Z-score was calculated for each fluorescence phenotype based on the block's controls.

Pyrosequencing to assay YFP copy number

We used a pyrosequencing assay to compare the relative number of YFP and CFP copies in diploid genomes. PCR amplification primers (Forward 5'-tgttgtcccaattttggttg-3', Reverse 5'-biotin-ccattcttttgtttgtcagca-3') were targeted to regions common to both coding sequences, but which flanked a position of dissimilarity. Colony PCR was performed as described above using the diploid patches on the mating plates. Using the PSQ96 (Biotage/Qiagen, Valencia, CA), an internal sequencing primer (5'-aggtcacaaattggaatac-3') common to both coding sequences was extended to a diagnostic position that differed between YFP and CFP. The relative abundances of YFP and CFP signal (normalized to the signal at the adjacent, invariant nucleotide) were observed to cluster distinctly around 50:50 and 67:33, corresponding to the ancestral 1-copy and duplicant 2-copy classes. All control samples of the ancestral genotype were found in the 50:50 cluster, as expected.

Transformation with mutant promoters

By introducing each mutation individually into the ancestral haploid genome, the effect of each observed promoter mutation could be assessed independent of the additional mutations on its original EMS-treated background. Mutant promoters, as well as the wildtype promoter control,

were amplified by PCR from their respective mutant strains, sequenced, and targeted to the transgenic *URA3* as described above. The intended promoter sequence of two independent transformants for each unique promoter was verified by colony PCR amplification and sequencing.

Comparison of engineered promoter strains

We used flow cytometry to compare the YFP fluorescence of strains with individual promoter mutations to positive and negative controls. Two representatives of each of eight genotypes were assessed: three were genotypes into which the mutations identified at positions -255, -240, and -140 (relative to the transcription start site identified in [68] had been introduced into the unmutagenized progenitor, the fourth, a strain in which a wildtype copy of the promoter had been introduced in parallel, and finally, the four mutant genotypes in which the promoter mutations were originally detected (including the two different isolates with the mutation at -255). All genotypes were cultured overnight in YPD. Aliquots of overnight cultures were diluted in arginine dropout media and cultured for two additional doublings before analysis by flow cytometry. Cultures were directly sipped by a C6 flow cytometer until 80,000 events had been recorded.

We compared the average fluorescence of genotypes containing site-directed promoter mutations to the reengineered wildtype control. After log transformation of FSC and YFP signal, FSC cutoffs were applied that culled the smallest (~10%) and largest (~15%) of cells. The fluorescence phenotype of each culture was defined as the median YFP/FSC of the remaining cells. If a significant difference was observed ($P<0.05$, MWW test), we inferred that the mutation affected YFP expression. We also tested the sufficiency of each mutation for the YFP

fluorescence phenotype of mutants in which it was originally observed, defined as a nonsignificant ($P>0.05$) result when site-directed promoter mutant cultures were compared to the respective original strain(s).

References

69. Brachmann CB, Davies A, Cost GJ, Caputo E, Li J, et al. (1998) Designer deletion strains derived from Saccharomyces cerevisiae S288C: A useful set of strains and plasmids for PCR-mediated gene disruption and other applications. Yeast 14: 115-132.

70. Cormack BP, Bertram G, Egerton M, Gow NAR, Falkow S, et al. (1997) Yeast-enhanced green fluorescent protein (yEGFP): a reporter of gene expression in Candida albicans. Microbiology 143: 303-311.

71. Gietz RD, Woods RA (2002) Transformation of yeast by lithium acetate/single-stranded carrier DNA/polyethylene glycol method. Methods Enzymol 350: 87-96.

72. R.Development.Core.Team (2008) R: A Language and Environment for Statistical Computing. Vienna, Austria: R Foundation for Statistical Computing.
